# Assessment of Soil-Heavy Metal Pollution and the Health Risks in a Mining Area from Southern Shaanxi Province, China

**DOI:** 10.3390/toxics10070385

**Published:** 2022-07-11

**Authors:** Rui Chen, Lei Han, Zhao Liu, Yonghua Zhao, Risheng Li, Longfei Xia, Yamin Fan

**Affiliations:** 1 School of Earth Science and Resources, Chang’an University, Xi’an 710054, China; ruichen@chd.edu.cn; 2 School of Land Engineering, Chang’an University, Xi’an 710054, China; lz13@chd.edu.cn (Z.L.); yonghuaz@chd.edu.cn (Y.Z.); 2021135016@chd.edu.cn (Y.F.); 3 Shaanxi Key Laboratory of Land Reclamation Engineering, Chang’an University, Xi’an 710054, China; 4 Key Laboratory of Degraded and Unused Land Consolidation Engineering, Ministry of Natural Resources of the People’s Republic of China, Xi’an 710054, China; 5 Shaanxi Provincial Land Engineering Construction Group, Xi’an 710075, China; lirisheng1209@163.com (R.L.); summer321xia@foxmail.com (L.X.)

**Keywords:** mining area, soil-heavy metal, pollution assessment, ecological risk, human health risk

## Abstract

Soil-heavy metal pollution in mining areas is one of the problems in the comprehensive treatment of soil environmental pollution. To explore the degree of soil-heavy metal pollution and the human health risk in mining areas, the contents of soil As, Cd, Cu, Cr, Hg, Ni, Pb, and Cr(VI) in an abandoned gold mining area were determined. The geoaccumulation index (I_geo_), single-factor pollution index (SPI), Nemerow comprehensive pollution index (NCPI), potential ecological risk index (PERI), and the human health risk assessment model were used to assess the pollution degree and the risk of soil-heavy metal pollution. Finally, the assessment results were used to provide remediation guidance. The results showed that (1) the average contents of As, Cd, Cr, Cu, Hg, and Ni in the mining area exceeded the background values of the soil elements. (2) The mining area was polluted by heavy metals to different degrees and had strong potential ecological hazards. (3) The total carcinogenic risk of heavy metals exceeded the health risk standard. The main components of pollution in the mining area were As, Cd, Cr, and Hg. Results from this study are expected to play a positive role in pollution treatment and the balance between humans and ecology.

## 1. Introduction

The exploitation of mineral resources not only promotes rapid economic growth but also threatens the surrounding ecological environment [1]. Soil-heavy metals have become a topic of focus all over the world because of their strong toxicity, high concealment, easy residue, and difficult treatment. The identification and environmental risk assessment of soil-heavy metal pollution characteristics in mining areas are the basis for regional soil-heavy metal pollution control [2]. In recent years, efforts have been made for the problem of soil-heavy metal pollution caused by mining. Chitsaz et al. [3] assessed soil pollution after copper mining in the Darreh Zereshk region of central Iran using Igeo and principal factor analysis and spatial distribution of elements. Liu et al. [4] used NCPI and Igeo to evaluate the levels of concentration of heavy metals in soil for potential ecological risk assessment in the Zhundong mining district. Wang et al. [5] combined SPI, NCPI, and a human health risk model to analyze soil Cr content in rural, urban, and suburban farmland soil. The spatial variability of soil pollution and geostatistical and statistical approaches to pollution are necessary and should be carried out at regular intervals [6,7]. Therefore, the continuous recording and monitoring of the pollution are established. In addition, data are emerging, which must be taken into account by policymakers [8]. On the other hand, determining and identifying the possible sources of pollution must be reliable; thus, the assessment of the problem becomes holistic and environmental management is more sustainable [9]. During a soil pollution investigation, the geoaccumulation index (I_geo_) [10], single-factor index (SPI) [11,12], Nemerow comprehensive index (NCPI) [13], potential ecological risk index (PERI) [14,15], and human health risk assessment [16,17,18] methods have been commonly used for the evaluation of pollution.

Previous studies have shown that land-use types have a certain impact on the migration and diffusion of soil-heavy metals [19]. For example, Chrastny et al. [20] showed that forest soils are much more affected with smelting processes, while agriculture soils are much more affected by downward metal migration. In addition, different land-use types have different reference values in pollution assessment [21]. Evaluating the degree of heavy metal pollution and potential ecological risk hazards based on different land use types is conducive to formulating targeted solutions, improving the quality of the soil environment, improving the living environment, and providing necessary support for the ecological environment management of mining areas. On this basis, to understand the pollution status and harm to the ecological environment and human health of soil-heavy metal pollution of different land use types in a mining area and its surroundings, soil samples were collected to determine the content of soil-heavy metals, and the pollution degree of soil-heavy metals in different land-use types in the mining area was analyzed by I_geo_, SPI, NCPI, and PERI to analyze the main pollution elements. Moreover, the human risk assessment model based on the heavy metal exposure pathway was used to evaluate the health risk to the surrounding population. Finally, the corresponding control measures were proposed according to the ecological environment and land-use types of the mining area and its surrounding areas. This study can provide a scientific basis and useful reference for remediating soil-heavy metal pollution in mining areas and residents’ health.

## 2. Materials and Methods

### 2.1. Study Area

**Figure 1:**
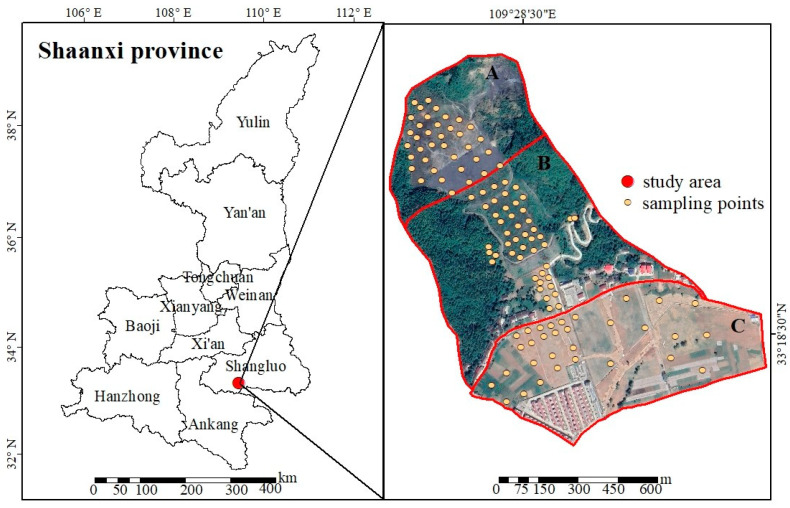
Location of the study area and sampling points (background image from Google Maps).

The city of Shangluo is located in the southeastern part of Shaanxi Province, China (Figure 1). It is between 108°34′20″–111°1′25″ E and 33°2′30″–34°24′40″ N and has a warm temperate climate. The annual average temperature is 7.8–13.9 °C; the annual average precipitation is 696.8–830.1 mm; the annual average sunshine duration is 1848.1–2055.8 h. The soil’s type is yellow cinnamon soil. A gold production company in the research area began operation in 1993; it ceased production after a dam failure in 2006. The comprehensive treatment project that adopted “microorganism + phytoremediation” technology for heavy metal pollution in farmland soil (area C) was launched by Shangluo Municipal Ecology Environment Bureau from 2016 to 2018. Even after several years, bare slag poses a serious threat to human health, and the research area is listed as one of the national key areas for heavy metal prevention and control. According to the Chinese Soil Environmental Quality Risk Control Standard for Soil Contamination of Development Land [22] and the Soil Environmental Quality Risk Control Standard for Soil Contamination of Agricultural Land [23], the study area can be divided into three subregions with different types (Figure 1). Area A is a pulp deposition area belonging to category 2 development lands where abandoned sludge accumulates with high heavy metal content. Area B is a hillside belonging to category 1 development lands, which is the buffer zone between the sludge deposition area and sloping farmland. Area C is farmland, belonging to agricultural land.

### 2.2. Field Investigation Sample Collection and Measurement

The soil samples were collected in November 2020. During sampling, sundries, such as large-grain gravel, weeds, and plant roots, were first removed from the soil. Wooden spades were then used to extract topsoil with a thickness of 0–20 cm. Diagonal sampling was used in five locations inside the quadrant. After uniformly mixing the collected materials from these five sites, the samples were quartered to reduce them to 1 kg and sealed in numbered polyethylene plastic bags. In total, 114 topsoil samples were collected, and their geographical coordinates were determined via real-time kinematic positioning with a precision of 1 cm.

Soil samples were dried indoors to a constant weight, and soil was then ground using a porcelain mortar, passed through a size-100 mesh, and stored. Soil samples were microwave-digested by using HNO_3_ (ρ = 1.42 g·mL^−1^) + HCl (ρ = 1.19 g·mL^−1^) + HF (ρ = 1.49 g·mL^−1^) + H_2_O_2_ (ω = 30%), and As, Cd, Cr, Cu, Ni, and Pb contents were measured using ICP-MS (Agilent 7700e ICP-MS) [24]. The detection limits were 0.5 mg·kg^−1^, 0.6 mg·kg^−1^, 1.0 mg·kg^−1^, 1.2 mg·kg^−1^, 1.9 mg·kg^−1^, 2.1 mg·kg^−1^, and 3.2 mg·kg^−1^. Hg content was measured by HNO_3_ (ρ = 1.42 g·mL^−1^) + HCL (ρ = 1.19 g·mL^−1^) heating digestion and atomic fluorescence spectrometry (Haiguang AFS-9760 atomic fluorescence spectrophotometer) [25]. The detection limit was 0.002 mg·kg^−1^. Soil Cr (VI) content was determined by alkaline digestion (30 g Na_2_CO_3_ and 20 g NaOH dissolved in water, diluted to 1 L) and flame atomic absorption spectrometry (Sherwood Scientific M420 flame spectrometry) [26]. The detection limit was 0.5 mg·kg^−1^. The soil’s pH value was determined by potentiometry [27]. All reagents used in this study were high-purity reagents, and Chinese national standard soil samples were used for quality control. In the sample determination, one sample was randomly selected from each 10 samples as a parallel sample for detection. When the error between samples and their parallel samples was not more than 5%, it was judged to be qualified.

### 2.3. Evaluation of Heavy Metal Pollution

#### 2.3.1. Geoaccumulation Index

I_geo_ is a pollution degree evaluation index proposed by Müller and is widely used to evaluate the metal pollution degree in water, ocean, and soil environments [28]. The calculation formula can be expressed as follows:

(1) $$I_{geo}=Log_{2}(\frac{C_{i}}{1.5B_{i}})$$

where *C_i_* (mg·kg^−1^) is the measured value of the target metal content in the soil, and *B_i_* (mg·kg^−1^) is the background value of the element. I_geo_ is divided into seven levels, as shown in Table 1.

#### 2.3.2. Single-Factor Pollution Index

The SPI describes the relationship between the measured value and the environmental limited standard value, which is used to evaluate a single pollution project. This method is simple and applicable to various types of pollution assessment [29]. The calculation formula is as follows:

(2) $$P_{i}=\frac{C_{i}}{S_{i}}$$

where *P_i_* is the single-factor pollution index, *C_i_* (mg·kg^−1^) is the measured value, and *S_i_* (mg·kg^−1^) is the reference standard value.

NCPI is a comprehensive index used to evaluate the level and degree of pollution in soil, water, and other environments under the action of various pollution factors [30]. The calculation formula is provided as follows:

(3) $$P_{\mathrm{com}}=\sqrt{\frac{{\left[\mathrm{ave}(P_{i})\right]}^{2}+{\left[\max\left(P_{i}\right)\right]}^{2}}{2}}$$

where *P*_com_ is the Nemerow pollution index, ave (*P_i_*) is the average value of a single pollution index of various pollution factors, and max (*P_i_*) is the maximum value of a single pollution index. NCPI can be divided into five levels, as shown in Table 2.

#### 2.3.3. Potential Ecological Risk Index

PERI is proposed by Hakanson to evaluate the ecological, environmental, and toxicological effects of heavy metals [31]. This method is widely used in related research [32] to reflect the impact of pollutants on the environment under specific environments and quantitatively classify the potential hazards of heavy metals.

The calculation formula of the potential ecological risk index of a single heavy metal element is as follows.

(4) $$E_{i}=T_{i}\times \left(C_{i}/S_{i}\right)$$

The calculation formula of the comprehensive potential ecological risk index of multiple heavy metals is as follows:

(5) $$RI=\sum E_{i}$$

where *T_i_* is the toxic response factor, *C_i_* (mg·kg^−1^) is the measured content of heavy metal *i*, and *S_i_* (mg·kg^−1^) is the reference ratio of heavy metal *i*. Table 3 shows the potential ecological risk index classification standard based on *E_i_* and *RI*.

### 2.4. Human Health Risk Assessment

The health risk assessment model of chemical substances recommended by the United States Environmental Protection Agency (U. S. EPA) [33] is used to assess the health risk of soil-heavy metal pollution in the study area. It mainly considers two heavy metal exposure pathways: the oral intake pathway and skin contact pathway.

1. The daily exposure of heavy metals through oral intake and skin contact is calculated as follows:
  (6) $$ADD_{i}=\frac{C_{i}\times IR_{ing}\times EF\times ED}{BW\times AT}10^{-6}$$
  (7) $$ADD_{i}=\frac{\;C_{i}\times SA\times AF\times ABS\times EF\times ED}{BW\times AT}10^{-6}$$
  where *C_i_* (mg·kg^−1^) is the measured content of heavy metals in the soil, and the other parameters are shown in Table 4.
2. The noncarcinogenic risk of a single pollutant is calculated as follows:
  (8) $$HQ_{ij}=\frac{ADD_{ij}}{R_{f}D_{ij}}$$
  where *R_f_D_ij_* (mg·(kg·d)^−1^) is the reference dose of heavy metal i under exposure pathway *j*, and the specific parameter values are shown in Table 5. An HQ value greater than 1 indicates that the pollutant has a certain noncarcinogenic risk; when it is less than 1, the noncarcinogenic risk is small or can be ignored.
3. The carcinogenic risk of a single pollutant is calculated as follows:
  (9) $$ILCR_{ij}=ADD_{ij}\times SF_{ij}$$
  where *SF_ij_* (kg·d·mg^−1^) is the carcinogenic tilt factor of heavy metal element *i* under exposure pathway *j*, and the specific parameter values are shown in Table 5.
4. Total noncarcinogenic risk:
  (10) $$HQ_{T}=\overset{m}{\underset{i=1}{\sum }}\overset{n}{\underset{j=1}{\sum }}HQ_{ij}$$
5. Total carcinogenic risk:
  (11) $$ILCR_{T}=\overset{m}{\underset{i=1}{\sum }}\overset{n}{\underset{j=1}{\sum }}ILCR_{ij}$$

If *ILCR* is less than 1.00 × 10^−4^, the heavy metal element does not have carcinogenic risk. Otherwise, the heavy metal element has carcinogenic risk [36].

### 2.5. Parameter Selection

The degree of soil-heavy metal pollution is mainly determined by comparison with a reference value. Therefore, selecting an appropriate parameter is an important part of reliable pollution evaluation. In this study, the filter values of development land and agricultural land were used as the reference value of the SPI, and the background value of Shaanxi Province was used as the reference value of the I_geo_ and potential ecological risk index. The main purpose here is to determine the excess of soil-heavy metals based on different land-use types and analyze the potential risk of heavy metals to the local ecological environment.

#### 2.5.1. Background Value of the Geoaccumulation Index

In the geoaccumulation index, B_n_ is the geochemical background value of the heavy metal element in the local area. According to the background values of soil elements in Shaanxi Province [37], B_As_ = 11.1 mg·kg^−1^, B_Cd_ = 0.094 mg·kg^−1^, B_Cr_ = 62.2 mg·kg^−1^, B_Cu_ = 21.4 mg·kg^−1^, B_Hg_ = 0.03 mg·kg^−1^, B_Ni_ = 28.8 mg·kg^−1^, B_Pb_ = 21.4 mg·kg^−1^, and B_Zn_ = 69.4 mg·kg^−1^ were selected as the background values of heavy metals.

#### 2.5.2. Toxicity Coefficient of the Potential Ecological Risk Index

In the process of health risk assessment, appropriate population parameters can improve the accuracy of the assessment results. In this study, the exposure dose of soil-heavy metals and population health risks in mining areas and surrounding areas were evaluated based on the partial parameter information of the rural population in Shaanxi Province according to the Handbook of Population Exposure Parameters in China [34] and the relevant parameters based on the technical guidelines for risk assessment of contaminated sites [35] (Table 5).

#### 2.5.3. Reference Value of the Single-Factor Pollution Index and Potential Ecological Risk Index

In the formulate calculating SPI, S_i_ refers to the reference standard value. For different regions, the S_i_ value can be selected as the filter value of the corresponding land-use type according to the soil environmental quality standard (Table 6), where the Cr in development lands is Cr(VI).

## 3. Results

### 3.1. Descriptive Statistics of Soil-Heavy Metals

The statistical results of the soil-heavy metal content in different subregions of the mining area are displayed in Table 7. All soil pH values in the study area were greater than 7.5, showing that the soil was weakly alkaline. The average contents of As and Hg in the three subregions and Cd in area C exceeded the filter value of the corresponding land-use types. Except for Pb and Zn, the average content of heavy metal pollutants in the three subregions exceeded the corresponding soil background value. These results indicate that the mining area and its surrounding soil were polluted by heavy metals to varying degrees. The average content of heavy metals was in the order of area A > area B > area C. The average As content in area A was 15.4- and 45.6-fold higher than that in area B and area C, respectively. The average Hg content in area A was 4.8- and 6.7-fold higher than that in area B and area C, respectively. The average coefficient of variation in each subregion followed the order of B > A > C. The coefficient of variation of As was the largest in area A and follow by area B and that of Hg was the largest in area C. The Kolmogorov–Smirnov test showed that concentrations of all elements are normally distributed with a statistical significance at the α = 0.05 level.

### 3.2. Evaluation of Heavy Metal Pollution in Soil

#### 3.2.1. Pollution Degree Analysis

1. Evaluation by the geoaccumulation pollution index

The background values of soil elements in Shaanxi Province were used as the reference values, and the I_geo_ method was applied to analyze the pollution degree in and near the mining area. The results are shown in Table 8. The three subregions were polluted to varying degrees by As, Cd, and Hg, with areas A and B being heavily to extremely heavily polluted, while Pb and Zn were not pollutants.

2. Evaluation by the single-factor pollution index

The calculation results of the SPI and NCPI of heavy metals in the mining area (Table 9) show that the soil As pollution in areas A and B reached a severe level and the Cd element pollution in area C reached a moderate level. The results of the Nemerow index suggest that area A was severely polluted, and areas B and C were slightly polluted.

#### 3.2.2. Potential Ecological Risk Assessment

The calculation results of the single potential ecological risk index and comprehensive potential ecological risk index (Table 10) indicate that Hg and Cd posed strong potential ecological hazard risks in the three subregions, As posed strong and moderate potential ecological hazard risks in areas A and B, respectively, and the other elements posed slight ecological hazard risks. On the whole, the potential comprehensive risk index of soil-heavy metals in the three subregions was 9350.97~61,796.91, with an average of 10,036.58, which posed a high ecological potential hazard risk.

### 3.3. Human Health Risk Assessment of Heavy Metals in Soil

#### 3.3.1. Exposure of Soil-Heavy Metals through Mouth and Skin Contact

**Figure 2:**
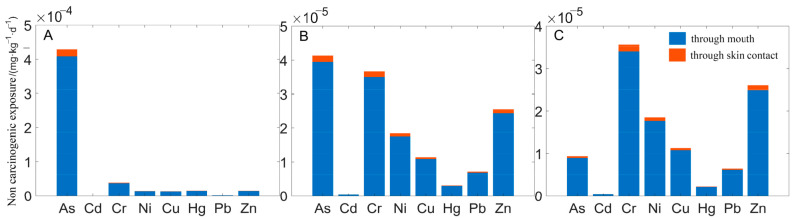
Noncarcinogenic exposure to heavy metals in soil. **A**, **B** and **C** represent area A, area B and area C, respectively.

**Figure 3:**
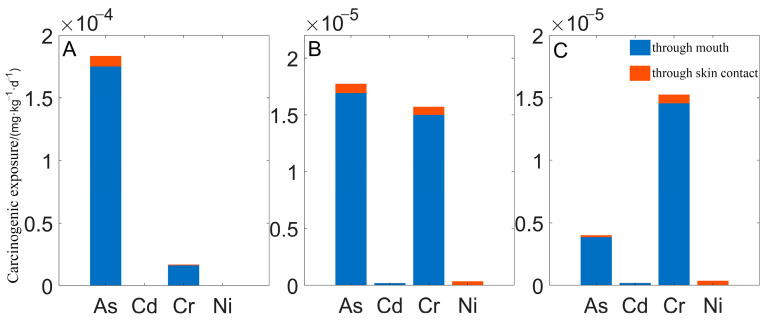
Carcinogenic exposure to heavy metals in soil. **A**, **B** and **C** represent area A, area B and area C, respectively.

The total noncarcinogenic exposure dose of heavy metals in soils of different land-use types (Figure 2) suggests that the noncarcinogenic exposure dose of heavy metals was between 10^−8^ and 10^−4^ mg·(kg·d)^−1^ through the mouth and between 10^−8^ and 10^−5^ mg·(kg·d)^−1^ through skin contact. The total carcinogenic exposure dose of heavy metals in the three subregions (Figure 3) show that the carcinogenic exposure dose of heavy metals was between 10^−7^ and 10^−4^ mg·(kg·d)^−1^ through the mouth and between 10^−9^ and 10^−6^ mg·(kg·d)^−1^ through skin contact.

#### 3.3.2. Human Health Risk Assessment Results of Soil Heavy Metals

**Figure 4:**
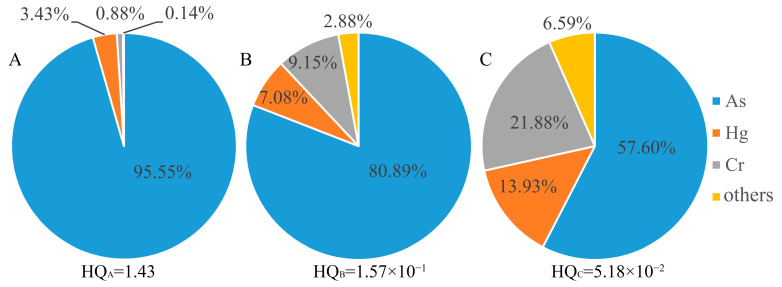
Noncarcinogenic risk contribution rate for oral intake of heavy metals in soil. **A**, **B** and **C** represent area A, area B and area C, respectively.

**Figure 5:**
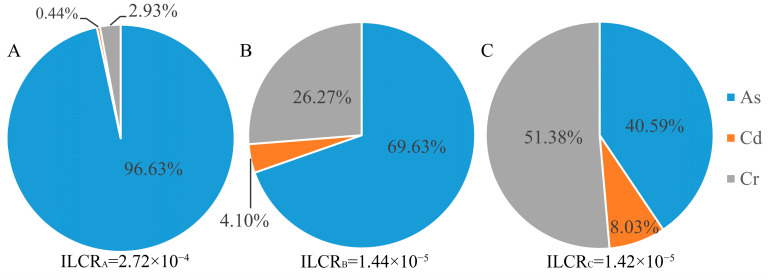
Carcinogenic risk contribution rate for oral intake of heavy metals in soil. **A**, **B** and **C** represent area A, area B and area C, respectively.

Figure 4 and Figure 5 show the noncarcinogenic risk and carcinogenic risk contribution rates caused by oral intake with soil-heavy metals. Among the three subregions, the noncarcinogenic risk of As through oral intake was greater than 1 in area A and less than 1 elsewhere. The total noncarcinogenic risks of areas A, B, and C were 1.43, 1.57 × 10^−1^, and 5.18 × 10^−2^, respectively, indicating that soil-heavy metals in the mining area posed a certain noncarcinogenic health risk to the surrounding population. The contribution rates of the noncarcinogenic risk of As through oral intake were 95.55%, 80.89% and 57.60%, accounting for most of the total noncarcinogenic risk. The carcinogenic risk of oral intake of the carcinogenic heavy metal elements ranged from 10^−6^ to 10^−4^, among which the contribution rate of As in area A was the highest, 96.63%.

**Figure 6:**
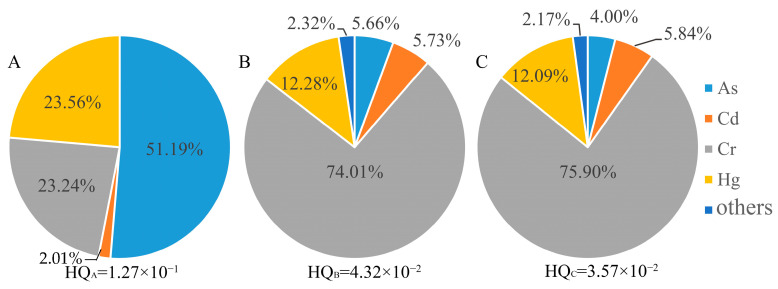
Noncarcinogenic risk contribution rate for skin contact with heavy metals in soil. **A**, **B** and **C** represent area A, area B and area C, respectively.

**Figure 7:**
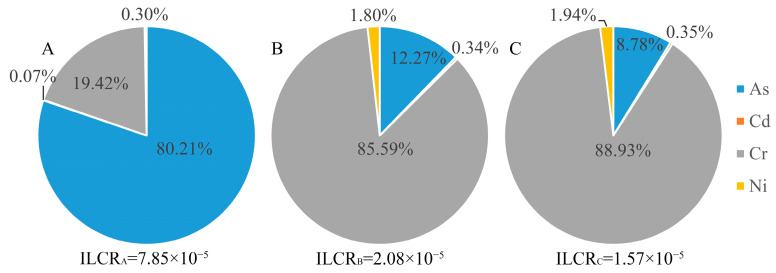
Carcinogenic risk contribution rate for skin contact with heavy metals in soil. **A**, **B** and **C** represent area A, area B and area C, respectively.

Compared with oral intake, the health risk of people exposed to heavy metals through skin contact was relatively small. The health risks of skin contact with As, Hg, and Cr in area A and Cr in areas B and C were higher than 1 × 10^−2^. The noncarcinogenic risk contribution rates of skin contact with Cr in area B and area C were as high as 74.01% and 75.09%, respectively (Figure 6). The carcinogenic risk of soil-heavy metals was between 10^−8^ and 10^−5^, and the carcinogenic risk rate of skin contact with As in area A was as high as 80.21% (Figure 7). In summary, the soil As pollution caused the greatest risk to human health in area A, area B, and area C, and Cr was the largest threat to human health through skin contact in area B and area C.

**Figure 8:**
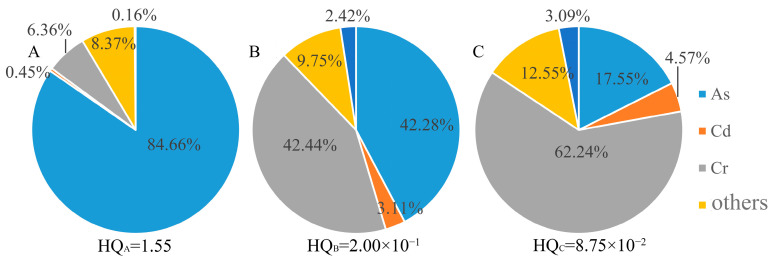
Noncarcinogenic risk contribution rate for soil-heavy metals. **A**, **B** and **C** represent area A, area B and area C, respectively.

**Figure 9:**
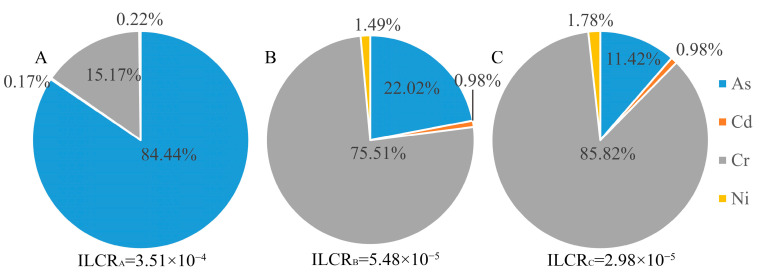
Carcinogenic risk contribution rate for soil-heavy metals. **A**, **B** and **C** represent area A, area B and area C, respectively.

Figure 8 and Figure 9 show the carcinogenic and noncarcinogenic risk contribution rates of soil-heavy metals through mouth and skin contact. The noncarcinogenic risks of heavy metals in the three subregions were 1.55, 2.00 × 10^−1^ and 8.75 × 10^−2^, respectively. The top three heavy metal elements were As, Hg and Cr. The sum of the multipath carcinogenic risks of the heavy metals in the three subregions was higher than the maximum acceptable carcinogenic risk value, which is 3.51 × 10^−4^, 5.48 × 10^−5^ and 2.98 × 10^−5^. Notably, the carcinogenic risks of As in areas A, B, and C were 3.26 × 10^−4^, 3.14 × 10^−5^, and 7.13 × 10^−6^, respectively. The highest contribution rate was in area A, up to 84.44%. The carcinogenic risk of Cr was 2.34 × 10^−5^, 2.18 × 10^−5^ and 2.12 × 10^−5^, respectively, in areas A, B, and C. The highest contribution rate of total carcinogenic risk in area C was 85.82%. 

## 4. Discussion

The NCPI and PERI are two common methods for evaluating heavy metal pollution [38]. In this study, the calculation results of the NCPI (Table 9) show that area A was severely polluted, and areas B and C were lightly polluted. The calculation results of the PERI (Table 10) indicate that the three subregions had a very high ecological risk. These indices showed some differences because their emphasis points are different. NCPI can amplify the impact of the highest content of pollutants among the sample points, while the PERI is used to differentiate the potential harm of different heavy metals to the ecosystem by weighting the toxicity response coefficient. Arsenic was the main pollutant in area A. The extremely high soil As content enlarged the characteristics of NCPI; thus, NCPI in area A was relatively high. When calculating PERI, the contents of Hg and Cd with high toxicity (high T_i_ value) in the three subregions were far beyond the local background value of the corresponding elements, and the As content in area A was high. Therefore, in general, strong potential ecological risks were found in the three subregions. Pollution assessment can reflect the potential harm of heavy metals to the ecological environment and provide the basis for improving the ecological environment. Human health risks can directly reflect the adverse health effects of heavy metals on exposed populations [39,40]. The results of the pollution assessment suggest that the main pollutants were As, Hg, and Cd. The results of the human health risk assessment indicate that As, Cr, Hg, and Cd had a high contribution rate to the carcinogenic and noncarcinogenic risks of the population in the study area (Figure 6 and Figure 7). This finding is due to the strong carcinogenicity of Cr under skin exposure; thus, the carcinogenic tilt factor SF_ij_ of Cr is higher [41]. Different evaluation methods have different emphases, so multiple evaluation indices should be comprehensively considered when evaluating the degree of heavy metal pollution to obtain more objective results.

The risk control ability of heavy metal pollution in the soil of mining areas is relatively weak. Many pollutants accumulate and diffuse in the soil, resulting in an increase in heavy metal content in the soil around the mining area and a decrease in soil quality, which seriously threatens the regional ecological environment and human health. Previous studies have shown that the heavy metal content in a mining area and surrounding soil exceeds the local background value and has a certain accumulation [42,43]. The potential sources of the soil-heavy metals can be determined by I_geo_ [21]. In this study, the contents of As, Cd, and Hg in the soil of the three sub-regions were seriously polluted, which were far higher than the local background values. This indicates that most of soil As, Cd, and Hg originated primarily from processing the ores and the disposal of tailings and high metal wastewaters around the mines [44]. Soil Cr, Cu, Ni, Pb, and Zn were unpolluted to lightly unpolluted, and with low coefficient deviation, indicating that the source was parent rocks and affected by human activities to some extent, for example, combustion of fuels used during processing is a potential source of Ni, Cr, and Cu in soils [45]. Area A was mainly composed of exposed mineral pulp, and the pollution degree was the highest. The pollution sources of areas B and C were pulp leakage caused by dam breaks. On the one hand, in the long-term erosion process of rain and sand, the soil interacted with the flow of water, resulting in heavy metal pollution in the surrounding soil. On the other hand, exposed pulps and soils with high concentrations of heavy metals can also be suspended in the atmosphere as dust and land in the periphery with the wind, endangering the health of agricultural land and the surrounding residents at low altitudes downwind (area C). In addition, the use of Hg-containing agricultural agents, such as ethyl mercuric chloride and phenylmercuric acetate, can also lead to an increase in Hg content in agricultural land [46].

Based on these findings, it is necessary to regularly detect and evaluate the heavy metal content and health risks in a mining area and its surrounding soil and take certain control measures [8]. In our study, area A, as the source of pollution, has the highest pollution degree and is in the upwind direction at high altitude, which poses a serious threat to areas B and C at low altitude. Therefore, the focus of area A is to prevent the diffusion of heavy metal pollution. Despite 15 years of natural weathering, area A still has a seriously excessive soil-heavy metal content, and strengthening the control measures is urgently needed. Because of the long-term accumulation of mineral pulp and a thick sludge layer, improved soil imported from other locations cannot be easily implemented and easily causes secondary pollution. Thus, chemical and biological remediation methods can be combined for treatment. First, in situ remediation should be carried out by chemical methods and then the contaminated site can be treated by phytoremediation and microbial remediation. The remediation plants should possess characteristics that include adaptation to the local environmental conditions and tolerance to a high concentration of metal pollutants [47]. Area B is the transition zone between polluted fields and farmland, where the pollution degree is low and the diffusion of contaminated soil to area C is possible. Therefore, the focus of treatment can be placed on blocking the spread of pollution from area A to area C. Plants can limit the dispersal of heavy metals by surface runoff and wind and, reduce entry into aquifers will be controlled [48]. Considering that area B is a hillside and the soil layer is thin, a practical method can be planting shrubs and grasses in this area to prevent the diffusion of contaminated soil to area C through scouring rainwater. Area C comprises farmland with a low pollution degree but still has potential ecological risks and human health risks. Suggestions for using improve soil imported from other places and combining soil amendments (e.g., organic, inorganic, and minerals) to quickly control heavy metal pollution in farmland soil surfaces are recommended. Tang et al. [49] found that the combined application of amendments improved the features of contaminated soil and reduced the availability of heavy metals more effectively. Then, selecting suitable crop species can not only ensure the safety of the regional ecological environment but also bring economic benefits.

## 5. Conclusions

The heavy metal pollution of soil in metal mining areas has been a focus of attention all over the world, and it is a focus of research for scholars. In this research, we analyzed the characteristics of soil-heavy metal pollution in the mining area by comparing various indicators and assessing human health risk; the main conclusions are as follows: (1) The average soil As, Cd, Cr, Cu, and Hg contents in the study area exceeded the corresponding soil background values in the city of Shangluo, Shaanxi Province, China. The soil-heavy metal content in area A was significantly higher than that in areas B and C and presented obvious spatial heterogeneity. (2) The calculation results of I_geo_, SPI, and NCPI demonstrate that the main pollutants in the three subregions of the mining area were As, Cd, and Hg. Area A was heavily polluted, and areas B and C were slightly polluted. (3) The calculation results of PERI suggest that Cd and Hg posed strong ecological risks in the three subregions. Among them, As in area A and B had a very high ecological risk. The comprehensive potential ecological risk index of the three subregions was very high. (4) The calculation results of the human health risk assessment indicate that the total noncarcinogenic risk and the total carcinogenic risk of heavy metals in the three subregions was in the order of area A > B > C. In summary, the pollution of As, Cd, Cr, and Hg in the mining area exceeds the acceptable risk, which is harmful to the surrounding ecological environment and people and needs to be used as the main pollution for the subsequent remediation of contaminated sites. These results can provide basic data for protecting and improving the soil environment in the research area, and the results provide useful references for soil environmental quality monitoring in mining areas.

## Figures and Tables

**Table 1:** Classification of I_geo_.

Classification	I_geo_	Pollution Degree
0	I_geo_ < 0	Unpolluted
1	0 ≤ I_geo_ < 1	Lightly polluted
2	1 ≤ I_geo_ < 2	Moderately polluted
3	2 ≤ I_geo_ < 3	Moderately to heavily polluted
4	3 ≤ I_geo_ < 4	Heavily polluted
5	4 ≤ I_geo_ < 5	Heavily to extremely polluted
6	I_geo_ ≥ 5	Extremely polluted

**Table 2:** Classification of the SPI and NCPI.

Classification	*P_i_*	Pollution Degree	*P*_com_	Pollution Assessment
I	*P_i_* ≤ 1	Clean	*P*_com_ ≤ 0.7	Clean (security)
II	1 < *P_i_*<2	Slight pollution	0.7 < *P*_com_ ≤ 1	Still clean (cordon)
III	2 < *P_i_* < 3	Moderate pollution	1 < *P*_com_ ≤ 2	Light pollution
IV	*P_i_* ≥ 3	Severe pollution	2 < *P*_com_ ≤ 3	Moderate pollution
V	-	-	*P*_com_ > 3	Severe pollution

**Table 3:** Classification of the potential ecological risk index.

Ecological Risk	Low	Moderate	Considerate	High	Very high
*E_i_*	<40	40–80	80–160	160–320	>320
*RI*	<150	150–300	300–600	-	>600

**Table 4:** Specific values of health risk assessment model parameters.

Symbol	Parameter Meaning	Value	Unit	References
IR_ing_	Daily soil intake	20	mg·d^−1^	[34]
SA	Exposed skin surface area	4350	cm^−2^	[35]
AF	Skin adherence factor	0.22	mg·cm^−2^·d^−1^	[34]
ABS	Dermal absorption factor	0.001	-	[35]
EF	Exposure frequency	350	d·a^−1^	[34]
ED	Exposure duration	30	a	[34]
BW	Average body weight	59.0	kg	[34]
AT (carcinogenic)	Average time	70 × 365	d	[34]
AT (noncarcinogenic)	Average time	30 × 365	d	[34]

**Table 5:** Carcinogenic and noncarinogenic factors of heavy metals under different exposure methods.

Item	Element	R_f_D_ij_ through Oral Intake	R_f_D_ij_ through Skin Contact	SF_ij_ through Oral Intake	SF_ij_ through Skin Contact
Carcinogenic heavy metals	As	3 × 10^−4^	3 × 10^−4^	1.5	7.5
	Cd	1 × 10^−3^	1 × 10^−5^	6.1	6.1
	Cr	3 × 10^−3^	6 × 10^−5^	0.5	20
	Ni	2 × 10^−2^	5.4 × 10^−3^	-	0.84
Noncarcinogenic heavy metals	Cu	4.2 × 10^−2^	1.2 × 10^−2^	-	-
	Hg	3 × 10^−4^	2.4 × 10^−5^	-	-
	Pb	3.5 × 10^−3^	5.25 × 10^−4^	-	-
	Zn	3 × 10^−1^	6 × 10^−2^	-	-

**Table 6:** Filter and control values of the heavy metal pollution risk for farmlands (pH > 7.5) and development lands (mg·kg^−1^).

	Farmlands		Development Lands			
Pollutant	Filter Values (mg·kg^−1^)	Control Values (mg·kg^−1^)	Filter Values (mg·kg^−1^)		Control Values (mg·kg^−1^)	
			Category 1 Lands	Category 2 Lands	Category 1 Lands	Category 2 Lands
As	25	100	20	60	120	140
Cd	0.6	4	20	65	47	172
Cr	250	1300	3.0	5.7	30	78
Cu	100	-	2000	18,000	8000	36,000
Hg	3.4	6	8	38	33	82
Ni	190	-	150	900	600	2000
Pb	170	1000	400	800	800	2500
Zn	300	-	-	-	-	-

**Table 7:** Soil-heavy metal concentrations in the mining area.

Area	Parameter	As	Cd	Cr	Cr(VI)	Cu	Hg	Ni	Pb	Zn	pH
A	Max (mg·kg^−1^)	1904.79	2.09	128.67	3.96	69.75	93.82	68.37	19.77	74.79	8.9
	Min (mg·kg^−1^)	678.11	1.17	94.23	0.73	27.54	6.13	43.32	3.71	32.62	8.0
	Mean (mg·kg^−1^)	1257.39	1.43	115.21	2.33	39.96	45.14	52.15	20.92	44.37	8.5
	Standard deviation	234.56	0.13	7.44	0.21	9.67	22.87	4	53	9.09	0.43
	Coefficient of variation	1.19	0.09	0.01	0.02	0.24	0.51	0.11	0.14	0.21	0.16
B	Max (mg·kg^−1^)	1229.13	2.13	136.52	2.33	46.35	38.88	76.37	35.83	95.80	8.6
	Min (mg·kg^−1^)	12.79	1.15	86.82	0.57	24.07	2.77	44.49	6.38	51.78	7.7
	Mean (mg·kg^−1^)	81.42	1.39	107.64	0.86	33.47	9.23	54.08	20.95	74.91	8.2
	Standard deviation	273.59	0.15	11.09	0.18	5.02	8.69	5.94	5.41	8.87	0.49
	Coefficient of variation	2.25	0.11	0.10	0.01	0.15	0.94	0.11	0.26	0.12	0.23
C	Max (mg·kg^−1^)	64.76	1.45	123.72	-	40.14	15.56	64.50	24.70	85.49	8.7
	Min (mg·kg^−1^)	15.66	1.19	86.83	-	25.13	3.25	46.19	15.59	62.19	7.5
	Mean (mg·kg^−1^)	27.52	1.34	104.51	-	33.21	6.66	54.32	18.87	76.46	8.4
	Standard deviation	19.55	0.08	9.48	-	4.06	2.99	4.12	1.72	5.93	0.47
	Coefficient of variation	0.31	0.06	0.09	-	0.12	0.45	0.08	0.09	0.08	0.08
Kolmogorov–Smirnov test		0.12	0.35	0.32	0.12	0.40	0.22	0.26	0.31	0.19	0.21

**Table 8:** Calculation results of I_geo_.

Item	As	Cd	Cr	Cu	Hg	Ni	Pb	Zn
A	6.24	3.34	0.30	0.32	9.97	0.27	−0.42	−1.23
Pollution degree	Extremely	Heavily	Lightly	Lightly	Extremely	Lightly	Unpolluted	Unpolluted
B	2.29	3.30	0.21	0.06	7.68	0.32	−0.62	−0.47
Pollution degree	Moderately to heavily	Heavy	Lightly	Lightly	Extremely	Lightly	Unpolluted	Unpolluted
C	0.72	3.25	0.16	0.05	7.21	0.33	−0.77	−0.45
Pollution degree	Lightly	Heavily	Lightly	Lightly	Extremely	Lightly	Unpolluted	Unpolluted

**Table 9:** Calculation results of the SPI and NCPI.

	SPI								NCPI
	As	Cd	Cr/Cr (VI)	Cu	Hg	Ni	Pb	Zn	
A	20.96	0.02	0.41	0.00	1.19	0.06	0.03	-	15.74 Severe
Pollution degree	Severe	Clean	Clean	Clean	Slight	Clean	Clean	Clean	
B	4.07	0.07	0.29	0.02	1.15	0.36	0.05	-	2.98 Light
Pollution degree	Severe	Clean	Clean	Clean	Slight	Clean	Clean	Clean	
C	1.10	2.23	0.42	0.33	1.96	0.29	0.11	0.25	1.79 Light
Pollution degree	Slight	Moderate	Clean	Clean	Slight	Clean	Clean	Clean	

**Table 10:** Calculation results of the potential ecological risk index.

	E_i_							RI	
	As	Cd	Cr	Cu	Hg	Ni	Pb	Zn	
A	1132.78	456.38	3.70	9.34	60,186.67	1.81	5.59	0.64	61,796.91
Risk degree	Very high	Very high	Low	Low	Very high	Low	Low	Low	Very high ecological risk
B	73.35	443.62	3.46	7.82	12,306.67	1.88	4.89	1.08	12,842.77
Risk degree	Moderate	Very high	Low	Low	Very high	Low	Low	Low	Very high ecological risk
C	24.79	427.66	3.36	7.76	8880.00	1.89	4.41	1.10	9350.97
Risk degree	Low	Very high	Low	Low	Very high	Low	Low	Low	Very high ecological risk

## Data Availability

Not applicable.

## List of Abbreviations

*ABS*	Dermal absorption factor
*ADD_i_*	The daily exposure of heavy metals through i pathway
*AF*	Skin adherence factor
*AT*	Average time
ave (*P_i_*)	The average value of a single pollution index of various pollution factors
*B_i_*	The background value of the element i
*BW*	Average body weight
*C_i_*	The measured value of the heavy metal element i in the soil
*ED*	Exposure duration
*EF*	Exposure frequency
*E_i_*	The potential ecological risk index of a single heavy metal element i
*HQ_ij_*	The carcinogenic risk of the heavy metal i under the exposure j
*HQ_t_*	The total noncarcinogenic risk
*Igeo*	The geoaccumulation index
*ILCR_ij_*	The carcinogenic risk of the heavy metal i under the exposure j
*ILCR_t_*	The total carcinogenic risk
*IR_ing_*	Daily soil intake
max (*P_i_*)	The maximum value of a single pollution index
NCPI, *P*_com_	Nemerow comprehensive pollution index
PERI	The potential ecological risk index
*R_f_D_ij_*	The reference dose of heavy metal i under exposure pathway j
*RI*	The comprehensive potential ecological risk index of multiple heavy metals
*SA*	Exposed skin surface area
*SF_ij_*	The carcinogenic tilt factor of heavy metal element i under exposure pathway j
*S_i_*	The reference value of heavy metal i
SPI, *P_i_*	The single-factor pollution index
*T_i_*	The toxic response factor
U. S. EPA	United States Environmental Protection Agency
